# Austrian multisociety consensus on metabolic dysfunction-associated steatotic liver disease: Austrian Society of Gastroenterology and Hepatology (ÖGGH), Austrian Society of Diabetology (ÖDG), Austrian Society of Obesity (ÖAG)

**DOI:** 10.1007/s00508-025-02617-4

**Published:** 2025-10-17

**Authors:** Mattias Mandorfer, Georg Semmler, Elmar Aigner, Alexander Bräuer, Johanna Maria Brix, Martin Clodi, Christian Datz, Maria Effenberger, Daniel Moritz Felsenreich, Bernhard Ludvik, Andreas Maieron, Markus Peck-Radosavljevic, Claudia Ress, Thomas-Matthias Scherzer, Harald Sourij, Lars Stechemesser, Herbert Tilg, Michael Trauner, Martin Wagner, Harald Hofer, Florian W. Kiefer, Peter Fasching, Michael Roden

**Affiliations:** 1 Division of Gastroenterology and Hepatology, Department of Medicine III, Medical University of Vienna, Währinger Gürtel 18–20, 1090 Vienna, Austria; 2 Vienna Hepatic Hemodynamic Lab, Division of Gastroenterology and Hepatology, Department of Medicine III, Medical University of Vienna, Vienna, Austria; 3 First Department of Medicine, University Clinic Salzburg, Paracelsus Medical University Salzburg, Salzburg, Austria; 4 5th Medical Department with Endocrinology, Rheumatology and Acute Geriatrics, Clinic Ottakring, Vienna Health Association, Vienna, Austria; 5 1st Medical Department with Diabetology, Endocrinology and Nephrology, Clinic Landstraße, Vienna Health Association, Vienna, Austria; 6 Karl Landsteiner Institute for Obesity and Metabolic Disorders, Vienna, Austria; 7 Clinical Division of Internal Medicine, Saint John of God Hospital, Linz, Austria; 8 Department of Internal Medicine, General Hospital Oberndorf, Teaching Hospital of the Paracelsus Medical University Salzburg, Oberndorf, Austria; 9 Division of Gastroenterology, Hepatology, Endocrinology and Metabolism, Department of Internal Medicine I, Medical University of Innsbruck, Innsbruck, Austria; 10 Division of Visceral Surgery, Department of General Surgery, Medical University of Vienna, Vienna, Austria; 11 Karl Landsteiner University of Health Sciences, Krems, Austria; 12 Department of Internal Medicine 2, Gastroenterology & Hepatology, University Hospital St, Dunant-Platz 1, 3100 St. Pölten, Austria; 13 Innere Medizin und Gastroenterologie (IMuG), Klinikum Klagenfurt, Klagenfurt am Wörthersee, Austria; 14 Department of Medicine I—Gastroenterology and Hepatology, University Hospital Wiener Neustadt, Wiener Neustadt, Austria; 15 Division of Endocrinology and Diabetology, Department of Internal Medicine, Medical University of Graz, Graz, Austria; 16 Division of Gastroenterology and Hepatology, Department of Internal Medicine, Medical University of Graz, Graz, Austria; 17 Research Unit for Translational Nuclear Receptor Research, Division of Gastroenterology and Hepatology, Medical University of Graz, Graz, Austria; 18 BioTechMed, Graz, Austria; 19 Department of Internal Medicine I, Gastroenterology and Hepatology, Rheumatology, Endocrinology and Diabetology, Klinikum Wels-Grieskirchen, Wels, Austria; 20 Clinical Research Institute for Inflammation Medicine, Johannes Kepler University, Linz, Austria; 21 Division of Endocrinology & Metabolism, Department of Medicine III, Medical University of Vienna, Vienna, Austria; 22 Department of Endocrinology and Diabetology, Medical Faculty and University Hospital, Heinrich Heine University, Düsseldorf, Germany; 23 Leibniz Institute for Diabetes Research at Heinrich Heine University, Institute for Clinical Diabetology, German Diabetes Center, Düsseldorf, Germany; 24 German Center for Diabetes Research (DZD e. V.), München-Neuherberg, Germany

**Keywords:** Non-alcoholic fatty liver disease, NAFLD, Non-alcoholic steatohepatitis, NASH, MetALD

## Abstract

This joint consensus document of the Austrian Societies of Gastroenterology and Hepatology (ÖGGH), Diabetology (ÖDG), and Obesity (ÖAG) is intended to provide practical guidance for the management of persons with metabolic dysfunction-associated steatotic liver disease (MASLD), including persons with combined metabolic dysfunction and alcohol-related steatotic liver disease (MetALD).

## Introduction

This joint consensus document of the Austrian Societies of Gastroenterology and Hepatology (ÖGGH), Diabetology (ÖDG), and Obesity (ÖAG) is intended to provide practical guidance for the management of persons with metabolic dysfunction-associated steatotic liver disease (MASLD), including persons with combined metabolic dysfunction and alcohol-related steatotic liver disease (MetALD). The management of persons with alcohol-related liver disease (ALD) is not within the scope of this document and should be based on respective international guidelines [1]. MASLD has previously been known as non-alcoholic fatty liver disease (NAFLD) and should be the term being used, while the term MetALD has been newly introduced [2]. Relevant aspects in the management of patients with compensated advanced chronic liver disease (cACLD) related to MASLD/MetALD are briefly outlined but further details should be derived from the respective Austrian (Billroth IV consensus and future versions) and international consensus statements (Baveno VII and future versions) [3, 4]. As such, the management of persons with decompensated cirrhosis is not covered, unless specifically stated. The certainty in the evidence and strength of recommendations was determined in analogy to the grading of recommendations, assessment, development, and evaluations (GRADE) framework (https://dev-bestpractice.bmjgroup.com/info/us/toolkit/learn-ebm/what-is-grade/) [5], if applicable:

- Very low (D): the true effect is probably markedly different from the estimated effect. / Any estimate of effect is very uncertain.
- Low (C): the true effect might be markedly different from the estimated effect. / Further research is very likely to have an important impact on our confidence in the estimate of effect and is likely to change the estimate.
- Moderate (B): the authors believe that the true effect is probably close to the estimated effect. / Further research is likely to have an important impact on our confidence in the estimate of effect and may change the estimate.
- High (A): the authors have a lot of confidence that the true effect is similar to the estimated effect. / Further research is very unlikely to change our confidence in the estimate of effect.

Strength of recommendation:

- Weak (2): indicates that engaging in a shared decision-making process is essential.
- Strong (1): suggests that it is not usually necessary to present both options.

## Definitions

**Fig. 1:**
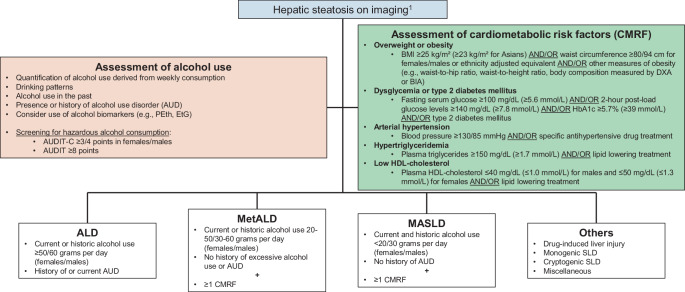
Diagnosis and work-up of steatotic liver disease (SLD) and its sub-entities. *ALD* alcohol-related liver disease, *AUD* alcohol use disorder, *BIA* bioimpedance analysis, *BMI* body mass index, *CMRF* cardiometabolic risk factors, *DXA* dual-energy X‑ray absorptiometry, *ETG* ethyl glucuronide, *MASLD* metabolic dysfunction-associated steatotic liver disease, *MetALD* metabolic dysfunction and alcohol-related steatotic liver disease, *PEth* phosphatidylethanol, *SLD* steatotic liver disease

**Fig. 2:**
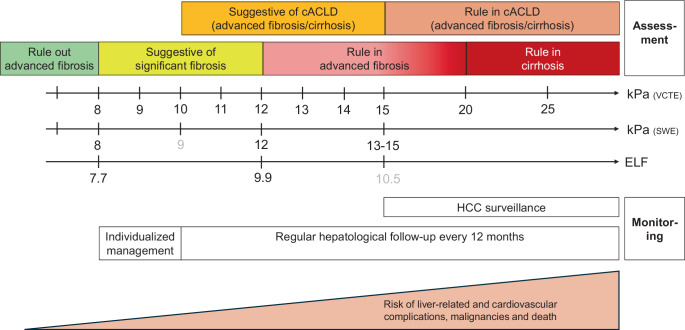
Interpretation of VCTE, SWE, and ELF and resulting monitoring recommendations. Numbers in grey indicate cut-offs supported by less evidence. *cACLD* compensated advanced chronic liver disease, *ELF* Enhanced Liver Fibrosis test, *HCC* hepatocellular carcinoma, *LSM* liver stiffness measurement, *SWE* shear-wave elastography, *VCTE* vibration-controlled transient elastography

- MASLD/MetALD are defined by the presence of steatosis on imaging (i.e., conventional B‑mode ultrasound or quantitative techniques, computed tomography, or magnetic resonance imaging [MRI]) or histology and at least one cardiometabolic risk factor (Fig. 1; [2, 6–8]).
- Metabolic dysfunction-associated steatohepatitis (MASH) is defined by hepatic steatosis and histological liver parenchymal injury, characterized by hepatocellular ballooning and lobular inflammation [7, 9].
- Histologically, hepatic fibrosis is staged as follows: no fibrosis (F0), mild fibrosis (F1), moderate/significant fibrosis (F2), advanced fibrosis (F3), and cirrhosis (F4). As non-invasive tests (NIT) are limited in their discriminative ability for individual stages, fibrosis should be classified by NIT as follows: significant fibrosis (≥ F2, i.e., presence of F2, F3, or F4), advanced fibrosis/cirrhosis (≥ F3, i.e., presence of F3 or F4), or cirrhosis (F4) [10, 11].
- Individuals with steatotic liver disease (SLD; i.e., umbrella term for persons with steatosis) consuming < 20/30 g alcohol per day (females/males) should be classified as MASLD, and those with current or historic alcohol consumption of 20-50/30-60g per day (females/males) as MetALD. Individuals with hepatic steatosis and either current or historic alcohol consumption > 50/60 g per day (females/males), or current diagnosis or history of alcohol use disorder should be classified as ALD [2, 6, 12, 13].
- Depending on the clinical context, SLD due to causes other than MASLD/MetALD or ALD (e.g., drug-induced liver injury, genetic metabolic disorders such as lysosomal acid lipase deficiency or hypobetalipoproteinemia, HIV-associated and other forms of lipodystrophy, endocrine diseases, and celiac disease) should be considered [6]. (*C1*).
- Steatosis may have resolved at the time of diagnosis of the most severe cases of SLD (i.e., cirrhosis). Thus, individuals may still be classified as MASLD/MetALD/ALD in the absence of steatosis in cases of high clinical suspicion [14]. (*C2*).
- Notably, the presence of fibrosis in persons with obesity and MASLD indicates clinical obesity, i.e., illness [15].

## Prevalence

- The prevalence of MASLD in the general population of Austria is estimated to lie between 35–55%, depending on the diagnostic modality for hepatic steatosis [16–19], 3–7% are estimated to have significant fibrosis (liver stiffness measurement, LSM by vibration-controlled transient elastography, VCTE ≥ 8 kPa) and ~ 1% advanced fibrosis, (LSM ≥ 12 kPa) [16, 19–22].
- About 3–5% of the global population are estimated to have MASH, but data from Austria are lacking [19].
- The prevalence of MASLD in individuals with type 2 diabetes mellitus (70–75%) and overweight/obesity (70–80%) is estimated to be higher, with consecutive higher rates of advanced fibrosis (~ 5–7%) [16, 17, 23, 24].

## Risk factors for disease progression

- Fibrosis stage determines the risk of liver-related events and thus subsequent management. Moreover, it is linked to cardiovascular events and other complications including malignancies [25–27]. (*A1*).
- Although the presence of MASH (i.e., steatohepatitis as evidenced by steatosis, ballooning degeneration of hepatocytes, and inflammation on histology) drives fibrosis progression, it cannot be reliably non-invasively assessed by monitoring transaminases, and specifically developed tests require further validation [6, 7, 28]. As the additional value for risk stratification on top of fibrosis stage is unclear [25, 29, 30], management decisions are currently guided by fibrosis stage [6, 7, 28]. (*B1*).
- Both metabolic dysfunction (especially type 2 diabetes mellitus and obesity) and alcohol have independent but also synergistic amplifying effects on disease phenotype and progression [31–33]. (*B1*).
- The following groups are at considerable risk for advanced fibrosis: type 2 diabetes mellitus, (abdominal) obesity, males > 50 years and postmenopausal women [6, 7, 16]. (*B1*).

## Alcohol consumption and assessment

- The current amount and drinking pattern as well as history of alcohol intake should be evaluated and documented in all individuals with suspected or diagnosed liver disease [6, 12, 13]. The daily amount of alcohol should be derived from the individual’s typical weekly consumption. (*B1*).
- Alcohol use disorder (AUD) should be evaluated by validated instruments (e.g., by AUDIT or AUDIT‑C, with a threshold for hazardous consumption of ≥ 8 points for AUDIT, ≥ 3 points for AUDIT‑C in females, and ≥ 4 points for AUDIT‑C in males) [6, 12, 13]. (*C1*).
- Alcohol intake may be evaluated by specific biomarkers (e.g., phosphatidylethanol (PEth) or ethyl glucuronide (EtG)) [6, 12, 13]. (*C2*).
- Complete alcohol abstinence may be encouraged in all persons with SLD, considering the harmful effects of alcohol consumption on overall health [6, 34]. (*A2*).
- Complete alcohol abstinence should be recommended in persons with liver fibrosis [6, 7]. (*B1*).

## Case finding

- Screening for SLD in the general population is not recommended [6, 7]. (*C1*)
- Case finding for liver fibrosis in SLD should be performed in the following at-risk groups [6, 7]:
  - Type 2 diabetes mellitus.
  - Obesity (abdominal/visceral) plus ≥ 1 additional cardiometabolic risk factors. Obesity is defined by BMI ≥ 30 kg/m^2^ and/or waist circumference ≥ 102 cm for men and ≥ 88 cm for women and/or other measures of visceral obesity [15].
  - Persistently elevated liver enzymes (i.e., aspartate aminotransferase, AST, alanine aminotransferase, ALT).
  - Hazardous/harmful alcohol consumption (alcohol consumption > 20/30 g per day for females/males or AUDIT-C ≥ 3/4 points or AUDIT ≥ 8 points). (*C1*).

## Fibrosis assessment and risk stratification

**Fig. 3:**
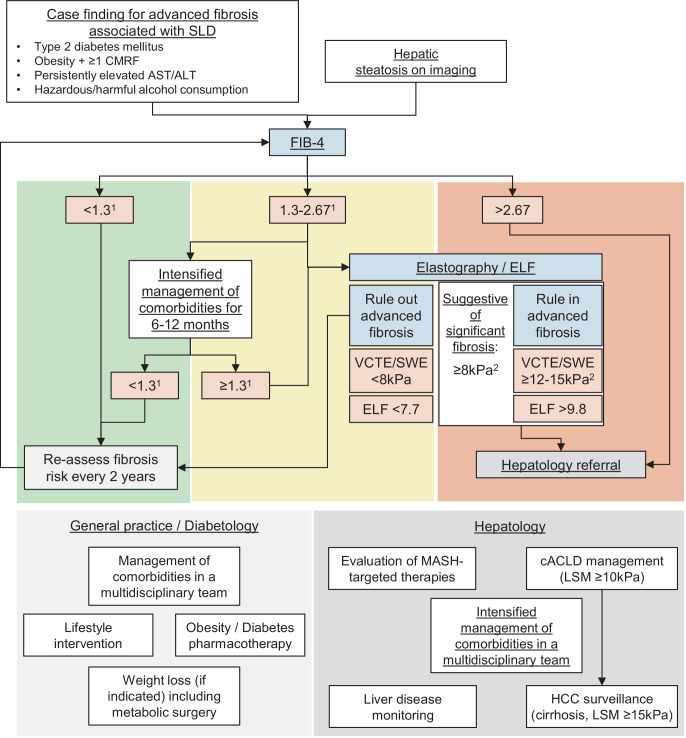
Algorithm for risk assessment in individuals with MASLD/MetALD, applicable for case finding of fibrosis associated with SLD. ^1^ Age-adjusted cut-off < 2 to rule out advanced fibrosis if age > 65 years. ^2^ If a false positive result is suspected, repeat after 2–4 weeks. *ALD* alcohol-related liver disease, *ALT* alanine aminotransferase, *AST* aspartate aminotransferase, *cACLD* compensated advanced chronic liver disease, *CMRF* cardiometabolic risk factors, *ELF* Enhanced Liver Fibrosis test, *HCC* hepatocellular carcinoma, *LSM* liver stiffness measurement, *MASH* metabolic dysfunction-associated steatohepatitis, *MASLD* metabolic dysfunction-associated steatotic liver disease, *MetALD* steatotic liver disease with metabolic-dysfunction and alcohol-related components, *SLD* steatotic liver disease, *SWE* shear-wave elastography, *VCTE* vibration-controlled transient elastography

- Early fibrosis detection and management of comorbidities may help to prevent its progression to cirrhosis and related complications [6]. (*C2*).
- NIT such as blood-based scores (e.g., fibrosis‑4, FIB‑4 score calculated as follows: age (years) × AST (U/L))/(platelet count (10^9^/L) × √ALT (U/L)) [35] or elastography should be used to estimate the probability of fibrosis and liver-related events in MASLD/MetALD, considering the clinical scenario (i.e., expected prevalence of fibrosis) and potential confounding factors [6, 7]. (*B1*).
- As NIT are more informative than AST, ALT, or gamma-glutamyl transferase alone, the latter should not be used for guiding management of people with MASLD/MetALD [6]. (*B1*).
- The following NIT thresholds rule out advanced fibrosis: FIB-4 < 1.3 (< 2 for age > 65 years), LSM < 8 kPa by VCTE or shear-wave elastography (SWE), or enhanced liver fibrosis (ELF) test < 7.7 [6, 7] (Fig. 2 and 3). (*B1*).
- LSM values ≥ 8 kPa by VCTE or SWE are suggestive of significant fibrosis while ≥ 10 kPa are suggestive of cACLD and indicate an increased risk of liver-related events. Values ≥ 12–15 kPa rule in advanced liver fibrosis, along with ELF > 9.8 [6, 7, 36] (Fig. 2 and 3). (*B1*).
- In cases of elevated LSM and recent excessive alcohol consumption (e.g., > 50/60 g per day or AUD) in combination with AST > 70 U/L or elevated bilirubin, elastography should be performed after 2–4 weeks of alcohol abstinence [8, 37]. The use of LSM can still reliably rule out advanced fibrosis in this situation [8, 37]. (*C2*).
- A sequential approach is recommended for identifying persons with liver fibrosis related to SLD (Fig. 3), applying a simple, nonproprietary blood-based NIT as first-line test (currently suggested: FIB-4) and, in case fibrosis cannot be ruled out, either elastography or ELF test [6, 7]. (*C1*).
- If elastography is easily accessible (e.g., in secondary or tertiary care), it may be applied as a first-line test. (*D2*).
- None of the available NIT to assess MASH can currently be recommended to guide clinical decision making [7, 8, 28, 38, 39]. (*B1*).

## Risk stratification regarding CSPH

- LSM < 15 kPa and platelet count ≥ 150 G/L rule out clinically significant portal hypertension (CSPH) in adults with MASLD/MetALD [3, 4, 40, 41]. (*B1*).
- LSM ≥ 25 kPa rules in CSPH in non-obese individuals with MASLD/MetALD [3, 4, 40, 41]. (*C1*).
- In obese persons, the ANTICIPATE ± NASH (LSM by VCTE, platelet count, ± BMI) or the NICER model (LSM and spleen stiffness measurement by VCTE, platelet count, BMI) may be applied to estimate the probability of CSPH [3, 4, 41, 42]. (*D1*).

## Steatosis assessment

- Given its broad availability, conventional B‑mode ultrasound is currently recommended as the primary diagnostic modality for establishing the diagnosis of SLD, although it has limited sensitivity for mild steatosis [6, 8]. (*A1*).
- MRI-based steatosis assessment by magnetic resonance spectroscopy or proton density fat fraction (MRI-PDFF) is currently considered the gold standard of steatosis assessment, but is limited by its cost and availability, and is therefore not recommended for broad clinical use [6, 10].
- Although SLD is defined by hepatic steatosis, its presence does not impact management of liver disease. Thus, steatosis assessment is currently only recommended when the goal is to establish a diagnosis of SLD and when this changes clinical management. (*C2*).

## Liver biopsy

- Liver biopsy should be reserved for the exclusion of other liver diseases [6]. (*A1*).
- Although required for the diagnosis of MASH, liver biopsy is not routinely indicated in this respect unless it changes clinical management [6]. (*A1*).
- Liver biopsy is not indicated for fibrosis assessment [6]. (*A1*).

## General management

- Comorbidities associated with MASLD/MetALD should be assessed/documented in all individuals and re-evaluated during follow-up to capture cardiometabolic risk [6] (*B1*):
  - Overweight/obesity.
  - Prediabetes and type 2 diabetes mellitus.
  - Arterial hypertension.
  - Dyslipidemia.
  - Obstructive sleep apnea syndrome.
  - Cardiovascular disease.
  - Chronic kidney disease.
  - In females: polycystic ovary syndrome.
- Individuals with MASLD/MetALD should be informed about the increased risk of extrahepatic malignancies and counselled about extrahepatic cancer screening recommendations [6]. (*B1*).
  Specifically, the following investigations are recommended:
  - Colonoscopy every 7–10 years starting at the age of 45 years or fecal immunochemical test (FIT)-based colorectal cancer screening every 2 years.
  - Biannual self-examination for skin lesions/cancer.
  - Immunization for human papilloma virus (HPV).
  - In females:
    - Yearly PAP smear and HPV testing every 3 years.
    - Mammography every second year starting at the age of 40 years.
  - In males:
    - Prostate cancer screening starting from the age of 45 years.
- In persons with type 2 diabetes, linkage to specialists in diabetes care and disease management programs is encouraged. (*B1*).
- Hepatology consultation is indicated in persons with NIT suggestive of significant fibrosis [6, 7, 38]. (*C1*).

## Nonpharmacological therapy

- Lifestyle modification is the foundation of MASLD/MetALD management [6]. (*A1*).
- A multidisciplinary approach is recommended to treat cardiometabolic comorbidities in MASLD/MetALD [6, 7]. (*B1*).
- Weight loss by lifestyle intervention (i.e., dietary, exercise, and behavioral therapy) is recommended in MASLD/MetALD [6, 7]. (*A1*).
- In adults with MASLD and overweight/obesity, weight management should aim at a sustained reduction of ≥ 5% to improve hepatic steatosis, 7–10% to improve hepatic inflammation, and ≥ 10% to improve liver fibrosis. Improvement of fibrosis is the key treatment goal, i.e., weight loss ≥ 10% should be intended [6, 43]. (*B1*). The same may apply to MetALD. (*C2*).
- Diet and exercise interventions may be also recommended in normal weight adults with MASLD to reduce liver fat, although there is currently no evidence regarding their impact on inflammation, fibrosis, or adverse liver-related outcomes [6, 44]. (*C2*).
- For adults with MASLD, improving diet quality (i.e., Mediterranean) as well as limiting the consumption of fructose (e.g., sugar-sweetened beverages) and ultraprocessed food is recommended [6, 45–48]. (*B1*). The same may apply to persons with MetALD, who may also be advised to abstain from alcohol. (*D2*).
- Physical activity and exercise (both aerobic and resistance; > 150 min/week of moderate or > 75 min/week of vigorous intensity) should be recommended and tailored to the individual’s preference and ability. This usually corresponds to 3–5 sessions of 30–60 min [6, 49–53]. (*A1*). The same may apply to persons with MetALD. (*C2*).
- In adults with MASLD/MetALD, nutraceuticals cannot be recommended as there is insufficient evidence regarding their health benefits, including their effectiveness in ameliorating hepatic inflammation/fibrosis or preventing adverse liver-related outcomes [6]. (*C1*).
- Observational studies have linked coffee consumption to improved liver health in MASLD [6, 7, 54–57]. (*C1*).

## General management in cACLD/cirrhosis

- Dietary and lifestyle recommendations should be adapted to the severity of liver disease, nutritional status, and the presence of sarcopenia/sarcopenic obesity in persons with cACLD/cirrhosis [6, 58, 59]. (*B1*).
- A high-protein diet (> 1.2–1.5 g/kg bodyweight/day) as well as a late evening snack are recommended for persons with cACLD/cirrhosis and sarcopenia/sarcopenic obesity [6, 58, 59]. (*B1*).
- In persons with cACLD/compensated cirrhosis and obesity, moderate weight reduction can be suggested, with an emphasis on high protein intake and physical activity to maintain muscle mass and reduce the risk of sarcopenia [6]. (*C2*).
- Further management should be based on the Billroth IV consensus or subsequent versions [3].

## Pharmacological treatment

- Individuals with MASLD/MetALD and evidence of significant liver fibrosis (i.e., ≥ 8 kPa) should be considered for MASH-targeted therapies (in particular, resmetirom [60] and semaglutide [61], once approved). (*D1*). Individuals with cACLD (LSM ≥ 10 kPa) have the highest/most urgent need for medicinal treatment due to their increased risk for adverse liver-related events [3, 4, 62, 63]. (*C1*).
- Resmetirom cannot be recommended for adults with a high probability of cirrhosis (i.e., ≥ 20 kPa) until phase 3 data establishing its safety and efficacy in this population are available (i.e., MASTRO-NASH OUTCOMES; NCT05500222) [6, 64]. (*D2*).
- Incretin-based therapies, currently indicated for type 2 diabetes as well as weight management in people with obesity or BMI ≥ 27 kg/m^2^ and comorbidities, should be used in people with MASLD/MetALD and evidence of significant liver fibrosis (i.e., ≥ 8 kPa), as they improve cardiometabolic outcomes and MASH [6, 38]. (*B1*). Specifically, the large phase 3 study on high-dose (2.4 mg) semaglutide demonstrated improvement of MASH and fibrosis [61]. Also, a smaller phase 2 study on tirzepatide indicated its efficacy in improving MASH and also found a reduction in fibrosis [65]. Similar findings have been obtained with survodutide [66], which has not been approved for type 2 diabetes, obesity, or MASH at the time of this consensus.
- In persons with MASLD/MetALD and evidence of significant liver fibrosis (i.e., ≥ 8 kPa), incretin-based therapies should be prescribed by specialists in internal medicine, endocrinology and/or diabetology, or gastroenterology and/or hepatology. (*D1*).
- From the hepatological perspective, pioglitazone is safe to use in persons without cirrhosis, however, it is not approved for and cannot be recommended as MASH-targeted therapy [6, 67]. (*B2*).
- Metformin is safe to use in MASLD/MetALD and should be used for its respective indication, namely type 2 diabetes, but cannot be recommended as MASH-targeted therapy [6]. (*C1*).
- Sodium-glucose co-transporter 2 (SGLT2) inhibitors are safe to use in MASLD/MetALD and should be used for their respective indications, namely type 2 diabetes, heart failure, and chronic kidney disease, but cannot be recommended as a MASH-targeted therapy at this point [6], although an investigator-initiated trial suggests efficacy in improving MASH and also found a reduction in fibrosis [68]. (*C1*).
- Insulin therapy should be used for its respective indication, type 1 and type 2 diabetes, but cannot be recommended as MASH-targeted therapy [69]. *(C1).*
- Despite limited evidence, individuals with MASLD/MetALD and a history of liver transplantation may be managed similarly. (*D2*).

## Pharmacological treatment in cACLD/cirrhosis

- While metformin can be used in adults with cACLD and preserved renal function (GFR > 30 ml/min), it should not be used in adults with decompensated cirrhosis [6]. (*C1*).
- Given the risk of hypoglycemia, sulfonylureas should be avoided in decompensated cirrhosis [6]. (*D1*).
- Incretin-based therapies can be used in adults with Child-Pugh class A cirrhosis [6, 70–72]. (*B1*).
- Hepatic impairment studies on semaglutide [70], tirzepatide [73], and survodutide [71] indicate that no pharmacokinetic-related dose adjustment is needed in mild to severe hepatic impairment (i.e., Child-Pugh classes A–C), although the potential risk of sarcopenia requires particular attention. (*D1*).
- Limited clinical experience indicates that incretin-based therapies may be used for weight management on the waiting list for liver transplantation [74], although the risk of sarcopenia requires particular caution. (*D2*).
- SGLT2 inhibitors are safe to use in people with Child-Pugh class A and B cirrhosis [6]. (*D2*).
- Statins should be used in cACLD/cirrhosis according to guidelines for reducing cardiovascular events [3, 4, 6]. (*B1*). In persons with Child-Pugh B/C, statins may be used at a lower dose due to an otherwise increased risk of rhabdomyolysis (e.g., 3% with simvastatin 40 mg [75]) and persons should be followed closely for muscle and liver toxicity. (*C1*) Simvastatin at max. 20 mg daily [75, 76] or atorvastatin 10 mg daily [77] have been shown to be safe in randomized controlled clinical trials including Child-Pugh B/C patients, while for rosuvastatin, only pharmacokinetic data are available, suggesting that a dose of 5 mg daily may be preferred [78].
- If CSPH is present, carvedilol should be used unless contraindicated [3, 4]. (*C1*).

## Metabolic/bariatric surgery

- In persons with MASLD without cirrhosis who have an indication for metabolic/bariatric surgery, techniques supported by the International Federation for the Surgery of Obesity and Metabolic Disorders (IFSO) may be considered, as metabolic/bariatric surgery has long-term benefits on liver health and may induce remission of type 2 diabetes and improvement of cardiometabolic risk factors [79]. (*C2*).
- In people with MASLD-related compensated advanced chronic liver disease/cirrhosis but without clinically significant portal hypertension, metabolic/bariatric surgery can be considered, but careful evaluation by a multidisciplinary team with experience in bariatric/metabolic surgery in this particular population is mandatory. Preoperative evaluation should follow the respective European Association for the Study of the Liver Clinical Practice Guidelines [80, 81]. (*D2*).
- Metabolic/bariatric endoscopic procedures require further evidence before being applied as a MASH-targeted therapy [6]. (*D2*).

## Natural history and general management

- As fibrosis determines outcomes in SLD, it should be considered the central parameter for liver disease monitoring and clinical decision-making [6, 7, 25, 29, 30]. (*A1*).
- Regression of fibrosis in persons with advanced fibrosis/cirrhosis is associated with a reduced risk of liver-related outcomes [62, 82–84]. (*B1*).
- Improvement in disease activity and resolution of steatohepatitis have been linked to fibrosis regression [85, 86]. (*B1*).

## Monitoring of comorbidities

- Comorbidities associated with MASLD/MetALD and cardiovascular risk should be assessed/documented in all individuals at diagnosis and re-evaluated during follow-up at regular intervals according to respective guidelines [6, 7]. (*A1*).
- During follow-up, special attention should be paid to diabetes mellitus and obesity due to their particularly strong associations with liver fibrosis progression [6, 7]. (*B1*)
- Extrahepatic cancer screening is recommended according to the respective guidelines [6, 7]. (*B1*)
- Alcohol consumption is an independent risk factor for the progression of SLD and should be assessed and documented in all individuals with suspected liver disease [6, 7]. (*A1*) While data on the impact, interval and modality of re-evaluation of alcohol consumption are lacking, we recommend re-assessment at regular intervals and according to the overall clinical context (e.g., when dynamics in NIT/laboratory parameters are observed), given the dynamic nature of alcohol consumption and associated harms [7]. (*D1*).

## Monitoring of fibrosis

- It is encouraged to repeat NIT to estimate disease progression or regression [62, 63, 83, 84]. (*C2*). Currently, there is insufficient evidence to define thresholds for a clinically significant change or response to treatment. (*D2*).
- Monitoring of fibrosis should preferably be done with the same NIT used for initial evaluation to assess changes over time. (*D1*).
- A gradual and consistent increase in NIT assessing fibrosis over time likely indicates worsening of fibrosis and increased risk of complications, while a decrease likely indicates improvement of fibrosis and a decreased risk of complications [62, 63, 83, 84]. (*C2*).
- In individuals with LSM values suggestive of cACLD (≥ 10 kPa), a regression to LSM < 10 kPa is associated with improved outcomes [62, 63, 83, 84]. (*C2*). There is currently limited evidence regarding the prognostic utility of dynamics in other NIT. (*D2*).
- NIT dynamics should be interpreted in the overall clinical context. (*D1*).
- Liver biopsy is not indicated for the purpose of fibrosis monitoring [6]. (*B1*).

## Monitoring intervals

- In persons with FIB-4 < 1.3 or in whom advanced fibrosis was ruled out (e.g., LSM < 8 kPa, ELF < 7.7), it is encouraged to repeat FIB‑4 every 2 years to reassess fibrosis probability [6, 7]. Other NIT may be repeated based on the overall clinical context/risk factors. (*D2*).
- In persons with cACLD (LSM ≥ 10 kPa), NIT should be repeated every 12 months [3, 4]. (*C1*).
- In people with LSM 8–10 kPa, there is currently insufficient evidence to recommend specific intervals for monitoring of liver disease but may be performed every 12 months based on the presence of risk factors. (*D2*).
- When monitoring a liver-directed treatment, it is recommended to repeat ALT/AST every 6 months and NIT for fibrosis every 12 months. (*D2*).

## Monitoring steatosis

- Regular monitoring of hepatic steatosis using ultrasound-based modalities is currently not recommended, as dynamics cannot be reliably assessed [7, 10] and as changes in the degree of hepatic steatosis do not impact clinical management. (*C1*).
- MRI-PDFF can be used to assess changes in hepatic steatosis, but it is not recommended for broad clinical use [6, 10] (*C1*).

## HCC surveillance

- HCC surveillance should be done according to respective guidelines and is not different to other chronic liver disease entities [6, 87]. (*B1*).
- HCC surveillance by ultrasound and alpha-fetoprotein is indicated in persons with SLD-related cirrhosis [6, 87]. (*B1*). LSM ≥ 15 kPa may be used as non-invasive cut-off to guide surveillance [88–90] (Fig. 3). (*C2*).
- HCC surveillance in other people than those with cirrhosis is currently not recommended due to cost effectiveness considerations [6, 87]. (*C2*).

## Abbreviations

A1–D2: Certainty of evidence and strength of recommendation according to the GRADE framework
ALD: Alcohol-related liver disease
ALT: Alanine aminotransferase
AST: Aspartate aminotransferase
AUD: Alcohol use disorder
AUDIT: Alcohol use disorders identification test
AUDIT‑C: Alcohol use disorders identification test-consumption
BIA: Bioimpedance analysis
BMI: Body mass index
BW: Body weight
cACLD: Compensated advanced chronic liver disease
CMRF: Cardiometabolic risk factors
CSPH: Clinically significant portal hypertension
DXA: Dual-energy X‑ray absorptiometry
ELF: Enhanced liver fibrosis test
EtG: Ethyl glucuronide
FIB‑4: Fibrosis‑4 score
FIT: Fecal immunochemical test
GFR: Glomerular filtration rate
GRADE: Grading of recommendations, assessment, development, and evaluations
HCC: Hepatocellular carcinoma
HPV: Human papilloma virus
IFSO: International Federation for the Surgery of Obesity and Metabolic Disorders
LSM: Liver stiffness measurement
MASH: Metabolic dysfunction-associated steatohepatitis
MASLD: Metabolic dysfunction-associated steatotic liver disease
MetALD: Metabolic dysfunction and alcohol-related steatotic liver disease
MRI: Magnetic resonance imaging
MRI-PDFF: Magnetic resonance imaging-proton density fat fraction
NAFLD: Non-alcoholic fatty liver disease
NIT: Noninvasive test
ÖAG: Österreichische Adipositas Gesellschaft/Austrian Society of Obesity
ÖDG: Österreichische Diabetes Gesellschaft/Austrian Society of Diabetology
ÖGGH: Österreichische Gesellschaft für Gastroenterologie und Hepatologie/Austrian Society of Gastroenterology and Hepatology
PEth: Phosphatidyl ethanol
SGLT2: Sodium/glucose cotransporter 2
SLD: Steatotic liver disease
SWE: Shear-wave elastography
VCTE: Vibration-controlled transient elastography
